# Prognostic characteristics and drug sensitivity analysis of hepatocellular carcinoma based on histone modification-related genes: a multi-omics integrated study revealing potential therapeutic targets and individualized treatment strategies

**DOI:** 10.3389/fphar.2024.1489469

**Published:** 2024-11-08

**Authors:** Ping Sun, Zheng Ding, Juan Chen, Kezhen Ou, Dianjie Zhou, Rui Li, Tianxiang Gu, He Sun, Ying Cheng

**Affiliations:** ^1^ Department of Organ transplantation and Hepatobiliary, The First Affiliated Hospital of China Medical University, Shenyang, Liaoning, China; ^2^ Key Lab Organ Transplantation of Liaoning Province, Shenyang, Liaoning, China; ^3^ Department of Cardiac Surgery, The First Affiliated Hospital of China Medical University, Shenyang, Liaoning, China; ^4^ Department of Medical Oncology, The First Affiliated Hospital of China Medical University, Shenyang, Liaoning, China

**Keywords:** histone modification, HCC, Hepatocellular carcinoma, machine learning, prognosis model, drug sensitivity

## Abstract

**Background:**

Hepatocellular carcinoma (HCC) ranks among the most prevalent and lethal malignancies worldwide. Histone modifications (HMs) play a pivotal role in the initiation and progression of HCC. However, our understanding of HMs in HCC remains limited due to the disease’s heterogeneity and the complexity of HMs.

**Methods:**

We integrated multi-omics data from multiple cohorts, including single-cell RNA sequencing, bulk RNA sequencing, and clinical information. Weighted gene co-expression network analysis (WGCNA) and consensus clustering were employed to identify histone-related genes. We developed a histone modification-related signature (HMRS) using 117 machine learning methods. Comprehensive analyses of molecular characteristics, immune landscape, and drug sensitivity associated with the HMRS were performed.

**Results:**

Through integrative analysis, we defined 110 histone-related genes and identified 45 HCC-HM-related genes (HCC-HMRgenes). The HMRS demonstrated robust prognostic value across multiple cohorts. Patients with high HMRS scores exhibited distinct genomic alterations, including higher tumor heterogeneity and TP53 mutations. The high-risk group showed enrichment in cell cycle, DNA repair, and metabolic pathways. Immune landscape analysis revealed significant differences in immune cell infiltration and pathway activities between high- and low-risk groups. Drug sensitivity prediction suggested potential therapeutic strategies for different risk groups.

**Conclusion:**

Our study provides a comprehensive understanding of HMs in HCC and establishes a robust prognostic signature. The HMRS not only stratifies patients into distinct risk groups but also offers insights into underlying molecular mechanisms, immune characteristics, and potential therapeutic strategies, paving the way for personalized medicine in HCC.

## 1 Introduction

Hepatocellular Carcinoma (HCC) ranks as one of the most prevalent and lethal malignancies worldwide, being the third leading cause of cancer-related deaths globally, with an average 5-year survival rate below 15% (Xia et al., 2022; Llovet et al., 2024). Alarmingly, the incidence of primary liver cancer is projected to rise by 55% by 2040 (Rumgay et al., 2022). This grim outlook is largely attributed to the complexity of its pathogenesis and limited treatment efficacy. While early-stage HCC can be treated through surgical resection, local ablation, or liver transplantation, the majority of patients are diagnosed at advanced stages. Current treatment modalities for advanced HCC primarily include local interventional therapies and systemic pharmacological interventions. Systemic drug therapies mainly comprise anti-angiogenic targeted therapies and immunotherapies, with the highest objective response rate (ORR) reaching only 36% (NCT03006926) (Finn et al., 2020). Consequently, there is an urgent need to elucidate the cellular mechanisms underlying HCC development to identify novel and effective therapeutic targets.

Epigenetic mechanisms form the foundation of the liver’s capacity to coordinate and regulate its regenerative abilities and adapt to rapidly changing environments, a unique feature among mammalian solid organs (Wilson et al., 2017; Michalopoulos and Bhushan, 2021). In recent years, epigenetic regulation, particularly histone modifications (HMs), has garnered increasing attention for its pivotal role in HCC initiation, progression, and treatment resistance. HMs primarily include acetylation, methylation, phosphorylation, ubiquitination, SUMOylation, ADP-ribosylation, and biotinylation. These modifications influence every aspect of HCC by manipulating the expression of oncogenes and tumor suppressor genes. Numerous studies have demonstrated that histone acetylation (Xia et al., 2022), methylation (Charidemou et al., 2023), and other modifications are associated with HCC occurrence, metastasis, angiogenesis, metabolism, apoptosis, immune homeostasis, and signaling pathways. For instance, Chen et al.'s research elucidated one pathway through which histone methylation affects HCC: the demethylase KDM4B may indirectly mediate miR-615-5p CpG demethylation through H3K9 (lysine nine on histone H3) demethylation. The absence of KDM4B promotes CpG methylation in the miR-615-5p promoter region, leading to decreased miR-615-5p expression. This, in turn, relieves miR-615-5p′s suppression of the oncogene RAB24, ultimately resulting in RAB24 overactivation and promoting HCC cell growth, migration, invasion, and adhesion (Chen et al., 2017). However, due to the heterogeneity of HCC and the complexity of HMs, our understanding of their comprehensive role and clinical significance in HCC remains limited and fragmented.

With the rapid development of high-throughput sequencing technologies and proteomics methods, we can now capture dynamic changes in HMs at the genome-wide level. These technological advancements provide unprecedented opportunities to systematically study HMs patterns and their functional significance in HCC. Meanwhile, the complexity and multidimensionality of these large-scale datasets require advanced computational methods to mine meaningful biological insights, and machine learning methods have shown great potential in deciphering complex biological problems.

This study aims to utilize various machine learning computational frameworks to systematically identify and analyze histone modification-related multi-omics features in HCC. By integrating genomics, transcriptomics, and proteomics data, we have revealed all key molecules and pathways of HMs affecting HCC occurrence and treatment response. This multi-omics integration approach not only provides a comprehensive understanding of epigenetic regulation in HCC but also identifies new diagnostic biomarkers and therapeutic targets, potentially transforming the challenging landscape of liver cancer treatment through epigenetic-targeted therapies. Additionally, this study combines patient follow-up data to construct an HCC risk prediction model based on HMs features. This model improves the accuracy of HCC prognosis assessment, supporting individualized treatment decisions. In this way, we aim to advance precision medicine for HCC, aligning with the current trend of 3P (Predictive, Preventive, Personalized) medicine.

## 2 Materials and methods

### 2.1 Data source

In this study, we primarily utilized data resources from three databases. First, we extracted gene expression data and corresponding survival information for 371 HCC samples from The Cancer Genome Atlas (TCGA) database (https://portal.gdc.cancer.gov/) (Tomczak et al., 2015). Second, we accessed HCC datasets GSE112271 and GSE14520 from the Gene Expression Omnibus (GEO) database (https://www.ncbi.nlm.nih.gov/geo/). GSE112271 includes single-cell RNA sequencing data from 7 HCC samples, while GSE14520 contains tissue sequencing data from 242 HCC samples with survival information. Additionally, we incorporated tissue sequencing data from 445 HCC samples with prognostic data from the International Cancer Genome Consortium (ICGC) (https://dcc.icgc.org/) (Zhang et al., 2019).

During data processing, we first extracted data in Transcripts Per Million (TPM) format from STAR count data and clinical information. Subsequently, to stabilize variance and improve data normality, we normalized the data and applied a log2(TPM+1) transformation. In the final stage of data preprocessing, we retained only samples with both RNA sequencing data and complete clinical information for subsequent analysis.

Furthermore, histone-related genes were sourced from two origins: cancer-associated HMs reported by Füllgrabe et al. (Oncogene, 2011) (Füllgrabe et al., 2011), and genes with HMs relevance scores greater than 20 from the GeneCards database (https://www.genecards.org/).

### 2.2 Single cell analysis

This study employed the Seurat package for comprehensive analysis of single-cell RNA-seq data (Stuart et al., 2019). We initially read 10X Genomics format data from the GSE112271 dataset and performed quality control, including calculating the proportion of mitochondrial and ribosomal RNA. After data filtering and normalization, we used PCA and UMAP for dimensionality reduction and applied the Harmony algorithm to integrate different samples (Cristian et al., 2024). We then conducted clustering analysis and used the SingleR package for cell type annotation. To construct histone scores, we calculated histone gene set enrichment scores for each cell using the single-sample Gene Set Enrichment Analysis (ssGSEA) method. Based on these scores, we divided cells into high and low score groups and performed differential expression analysis using Seurat’s FindAllMarkers function. We also visualized the distribution of histone scores across different cell types using FeaturePlots and violin plots. This series of analyses not only revealed cellular heterogeneity in histone expression but also provided a foundation for further exploration of histone-related functions and regulatory mechanisms.

### 2.3 Weighted gene co‐expression network analysis (WGCNA)

This study employed Weighted Gene Co-expression Network Analysis (WGCNA) to thoroughly investigate the association between RNA-seq data and histone expression in the TCGA database (Langfelder and Horvath, 2008). We first calculated histone scores for each sample using the ssGSEA method as phenotype data for subsequent analysis. During data preprocessing, we performed sample clustering and outlier detection to ensure data quality. Subsequently, through soft threshold selection and network construction, we identified multiple gene co-expression modules. Further, we analyzed the relationship between Module Membership and Gene Significance of genes in these modules, providing a basis for identifying key regulatory genes.

### 2.4 Construction of prognostic features through integrated machine learning methods

This study employed various machine learning algorithms to construct prognostic models, including Random Survival Forest (RSF) (Ishwaran et al., 2008), Elastic Net (Enet) (Zou and Hastie, 2005; Cho et al., 2009), Stepwise Cox Regression (StepCox) (Liu et al., 2023), CoxBoost (Binder and Binder, 2015), Partial Least Squares Cox Regression (plsRcox) (Bertrand et al., 2022; Bertrand et al., 2014), SuperPC (Bair et al., 2006), Gradient Boosting Machine (GBM) (Ayyadevara and Ayyadevara, 2018), Survival Support Vector Machine (survival-SVM) (Van Belle et al., 2011), Ridge Regression (Arashi et al., 2021), and Lasso Regression (Ranstam and Cook, 2018). The TCGA dataset was used as the training set, with GSE14520 and ICGC datasets serving as validation sets. Data was first standardized, then models were constructed using each algorithm and evaluated on the validation sets. To enhance model stability, we also experimented with up to 117 algorithm combinations, such as RSF + CoxBoost and Lasso + GBM. The C-index was used to assess model discriminatory ability across datasets. Finally, C-index results for all models across different datasets were compiled into a heatmap, visually demonstrating each model’s predictive performance. By comparing the performance of different algorithms and their combinations, we aimed to identify the optimal prognostic prediction model. Subsequently, results and features were visualized based on model weights.

### 2.5 Survival analysis and nomogram construction

This study conducted a comprehensive analysis of the TCGA dataset, exploring relationships between risk scores, clinical features, gene expression, and survival outcomes. We processed clinical data, created pie charts comparing clinical features, compared risk scores across different T stages using violin plots, and produced stacked bar charts showing the proportion of clinical features in high- and low-risk groups. We also analyzed gene expression data and created heatmaps to display expression differences. Logistic regression was used to predict M stage, with ROC curves assessing predictive performance (Stoltzfus, 2011; Blanche and Blanche, 2019). Subsequently, we plotted Kaplan-Meier survival curves based on patient age and clinical stage, comparing survival differences between high- and low-risk groups.

To further enhance the model’s predictive accuracy and prognostic capability, we developed a nomogram combining histone and clinical features to quantify expected survival for HCC patients (Park, 2018). After identifying independent prognostic factors through univariate and multivariate Cox regression analyses, we constructed a nomogram based on multivariate Cox regression results, visually demonstrating each factor’s contribution to prognosis. Calibration curves were used to evaluate the model’s predictive accuracy. Decision curve analysis (DCA) assessed the model’s clinical application value (Fitzgerald et al., 2015). Additionally, we calculated the C-index to measure the model’s discriminatory power and plotted time-dependent C-index curves to compare long-term predictive capabilities of different predictors. Finally, we validated the model’s internal stability through Bootstrap resampling (Henderson, 2005). These methods comprehensively evaluated the prognostic model’s predictive accuracy, clinical utility, and stability, providing reliable evidence for its clinical application.

### 2.6 GSEA and GSVA functional enrichment analysis

This study continued to employ various bioinformatics methods to explore the relationship between gene expression patterns and prognostic risk. We used the limma package for differential expression analysis to identify differentially expressed genes between high- and low-risk groups (Ritchie et al., 2015). Subsequently, Gene Set Enrichment Analysis (GSEA) was used to explore functional pathways of differentially expressed genes, and Gene Set Variation Analysis (GSVA) was employed to quantitatively score pathway activity for each sample (Hung et al., 2012; Hänzelmann et al., 2013). We performed inter-group differential analysis on GSVA scores and created volcano plots to display significantly altered pathways. Additionally, we calculated correlations between GSVA scores and risk scores, presenting them visually through heatmaps. Finally, we conducted survival analysis on key pathways to identify those significantly associated with prognosis.

### 2.7 Mutation analysis

To further reveal the relationship between tumor mutation characteristics and prognostic risk, and to understand the biological basis of the risk score model, we employed various methods to analyze the relationship between tumor mutation characteristics and prognostic risk. Firstly, we used the maftools package (Mayakonda et al., 2018) to calculate the Mutant-Allele Tumor Heterogeneity (MATH) score for each sample and compared differences between high- and low-risk groups. Kaplan-Meier survival analysis was used to evaluate the association between MATH scores and patient prognosis. Subsequently, we performed stratified survival analysis combining MATH scores and risk scores to explore their joint predictive effect. Additionally, we conducted mutation landscape analysis for high- and low-risk groups separately, creating oncoplots to display the top 20 mutated genes. We also used the somaticInteractions function to analyze co-mutation and mutual exclusivity relationships between genes, revealing patterns of gene mutation interactions in different risk groups.

### 2.8 Immune characteristics analysis

In our study, to explore the relationship between immune cell infiltration in the HCC tumor microenvironment (TME) and histone modification-related signature (HMRS), we utilized the IOBR software package (Zeng et al., 2021) to assess ESTIMATE, CIBERSORT, and the infiltration of 28 immune cell types in HCC samples from TCGA. We used the ESTIMATE algorithm to evaluate stromal, immune, and comprehensive scores of tumor samples, comparing differences between high- and low-risk groups. Subsequently, we employed the ssGSEA method to score immune-related pathways and 28 immune cell types, and used the CIBERSORT algorithm to estimate the proportions of 22 immune cell types, thoroughly investigating differences in the immune microenvironment between risk groups (Chen et al., 2018). These analyses were visualized through box plots and heatmaps, clearly demonstrating immune characteristic differences between high- and low-risk groups. Furthermore, we conducted correlation analyses between characteristic genes and immune cells, as well as between risk scores and immune cells, presenting these complex relationships through correlation heatmaps. These multi-level, multi-faceted analyses not only revealed the complexity of the tumor immune microenvironment but also provided important insights into the immunological basis of the risk score model, laying a foundation for further immunotherapy research.

### 2.9 Significance of the HMRS in drug sensitivity

In this study, we utilized the Genomics of Drug Sensitivity in Cancer (GDSC) database (https://www.cancerrxgene.org/) to predict the sensitivity of high- and low-risk group samples to common anticancer drugs. This is one of the largest public resources in the field of pharmacogenomics, providing rich information on drug sensitivity and related genomics, crucial for discovering potential cancer treatment targets (Yang et al., 2013). To this end, we applied the pRRophetic software package (Geeleher et al., 2014) to construct cell line-based ridge regression models using drug information and gene expression data from the CGP2016 dataset, and then conducted predictive analysis for each possible drug. Using the pRRopheticPredict function, we predicted the half-maximal inhibitory concentration (IC50) values for each drug based on the gene expression profiles of tumor samples (Sebaugh, 2011). Subsequently, we combined the predicted drug sensitivities with our previously established risk score model to compare drug sensitivity differences between high- and low-risk groups. We used the Wilcoxon rank-sum test to assess the statistical significance of these differences and created box plots for drugs with significant differences using the ggplot2 package.

### 2.10 Experiment validation

To validate the biological significance of our HMRS model, we conducted further pathological verification on the top five genes with the highest weights in the model. First, we utilized the Human Protein Atlas (HPA) database (https://www.proteinatlas.org/) to compare the protein expression levels of these genes in pancreatic cancer tissues and adjacent normal pancreatic tissues (Uhlén et al., 2015). For genes lacking data in the HPA database, we performed laboratory validation.

We collected paired PDAC and adjacent pancreatic tissue samples from PDAC patients who underwent surgical resection at our center. Tissue samples were fixed in 4% paraformaldehyde and embedded in paraffin to create 4 μm thick sections. Standard immunohistochemistry (IHC) staining procedures were followed. Briefly, sections were deparaffinized, rehydrated, and underwent antigen retrieval in citrate buffer (pH 6.0). Endogenous peroxidase activity was blocked with 3% H2O2, and non-specific binding was blocked with 10% goat serum. Subsequently, sections were incubated overnight at 4°C with the corresponding primary antibodies. ASF1A antibody (1:1,000 dilution, Proteintech) was used. The next day, sections were incubated with HRP-labeled secondary antibodies for 1 h at room temperature. DAB was used for color development, followed by hematoxylin counterstaining. Staining results were independently evaluated by two experienced pathologists who were blinded to the clinical information.

All patients provided written informed consent, and this research protocol was approved by the Ethics Committee of the First Affiliated Hospital of China Medical University (approval number: KT20241,196).

## 3 Results

**FIGURE 1:**
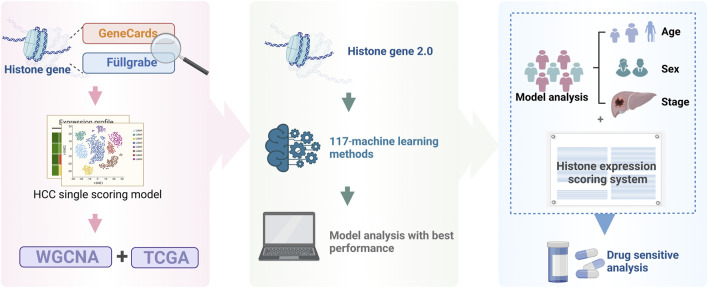
Study flowchart.

All analytical processes are illustrated in the flowchart (Figure 1).

### 3.1 Histone modification characteristics in single-cell transcriptomics

**FIGURE 2:**
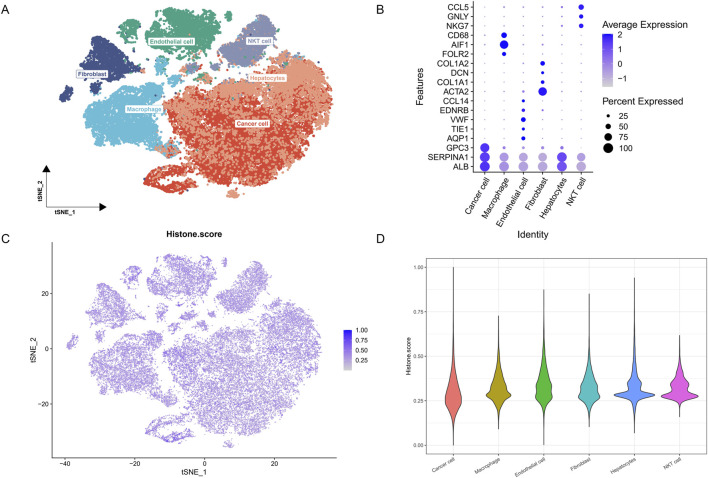
HMs characteristics in single-cell transcriptomics. **(A)** t-SNE plot showing the cell types identified by marker genes. **(B)** Dot plot showing the marker genes in each cell cluster. **(C)** The activity score of HMs in each cell. **(D)** The distribution of the histone score in different cell types.

To gain a deeper understanding of HMs characteristics across different cell types, we conducted a comprehensive analysis of single-cell transcriptome data. Utilizing t-SNE technology, we successfully identified and annotated six major cell clusters: endothelial cells, macrophages, hepatocytes, cancer cells, fibroblasts, and NKT cells, revealing the complex distribution pattern of multiple cell populations within the HCC microenvironment (Figure 2A). To further validate the accuracy of cell type annotations, we generated a dot plot displaying the expression of marker genes for each cell cluster (Figure 2B).

Subsequently, we calculated and visualized the HMs activity score for each single cell using the 110 histone-related genes we had constructed (Figure 2C). To more intuitively compare the differences in HMs activity among different cell types, we employed violin plots to illustrate the distribution of HMs scores across cell populations (Figure 2D). We discovered that HMs activity is ubiquitous across all cell types, albeit with a degree of heterogeneity.

Based on the overall cellular HMs activity, we categorized cells into high HMs and low HMs groups. By comparing these two groups, we identified 772 differentially expressed genes related to HMs (single cell HM-related DEGs), all derived from HCC single-cell data.

### 3.2 Identification of histone-related genes in HCC bulk RNA sequencing

**FIGURE 3:**
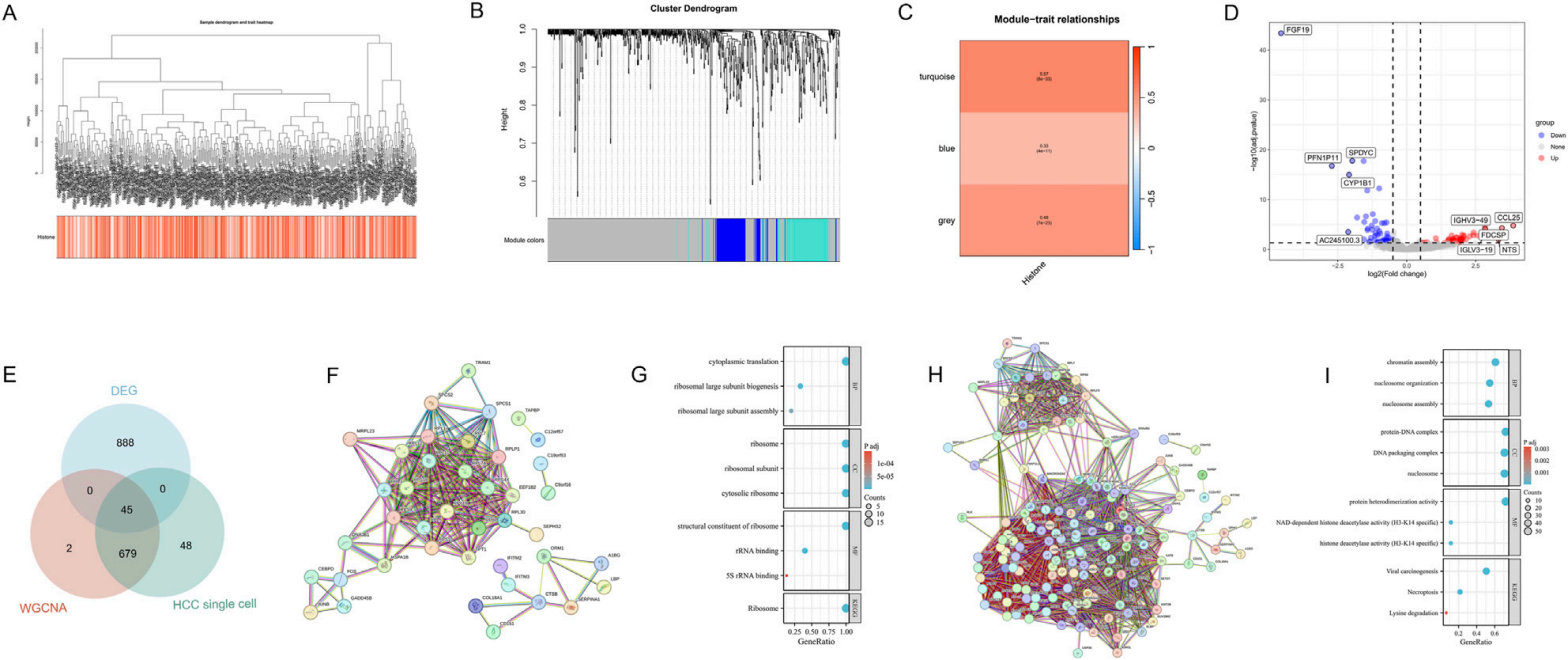
Identification and functional analysis of HCC-HMRgenes. **(A)** Dendrogram showing hierarchical clustering of TCGA-HCC samples. The bottom heatmap represents the HMs score for each sample. **(B)** Cluster dendrogram of the WGCNA analysis. **(C)** Module-trait heatmap showing the closely related modules to the HMs trait. **(D)** Volcano plot displaying differential analysis results between TCGA-HCC and normal samples, highlighting the top five up- and downregulated genes with the most significant expression changes. **(E)** Venn plot showing the intersecting genes between single-cell HM-related DEGs, HMs Module genes, and HCC DEGs. **(F)** PPI Network of 45 HCC-HMRgenes. **(G)** GO and KEGG Analysis Results of 45 HCC-HMRgenes. **(H)** PPI Network of updated HCC-HMRgenes related genes. **(I)** GO and KEGG Analysis Results of updated HCC-HMRgenes related genes.

We employed the WGCNA method to construct a hierarchical clustering dendrogram of TCGA-HCC samples (Figure 3A), illustrating the clustering relationships among samples. Additionally, the heatmap at the bottom of the figure visually presents the HMs scores for each sample, reflecting the relative activity of HMs characteristics within the samples. Further analysis of the sample clustering dendrogram (Figure 3B) and the module-trait heatmap (Figure 3C) revealed that the turquoise, grey, and blue modules were closely associated with HMs. These modules collectively encompass 726 genes, including 99 in blue, 493 in grey, and 134 in turquoise. To further narrow down the candidate gene pool, we identified differentially expressed genes (DEGs) between normal and HCC samples in the TCGA dataset, yielding HCC DEGs (Figure 3D). Building upon this, we intersected single-cell HM-related DEGs, HMs Module genes, and HCC DEGs, ultimately obtaining 45 intersecting genes, designated as HCC and histone-related genes (HCC-HMRgenes) (Figure 3E).

To explore the interactions among these 45 HCC-HMRgenes, we constructed a protein-protein interaction (PPI) network (Figure 3F). Subsequently, we performed GO and KEGG enrichment analyses (Figure 3G) to investigate the distribution of these 45 genes in biological processes (BP), cellular components (CC), and molecular functions (MF), as well as their potential roles in various biological pathways. Results indicated that in terms of BP, CC, and MF, the genes were primarily enriched in ribosome structure and function, as well as protein translation-related pathways. KEGG analysis revealed that the ribosome pathway exhibited the most significant and unique enrichment. This suggests that HMs may promote tumor progression by influencing ribosomal function and, consequently, protein synthesis. Next, we combined the previously defined 110 genes with these 45 HCC-HMRgenes and reanalyzed their interaction networks and enrichment to further investigate HMs mechanisms in HCC (Figures 3H, I). GO analysis results showed that chromatin structure and nucleosome organization are closely related to HMs in HCC, with histone deacetylation potentially playing the most crucial role. KEGG pathway analysis revealed that viral infection might promote HCC development by influencing HMs, while HMs also plays a role in regulating cell death.

### 3.3 Construction of prognostic signature based on integrated machine learning

**FIGURE 4:**
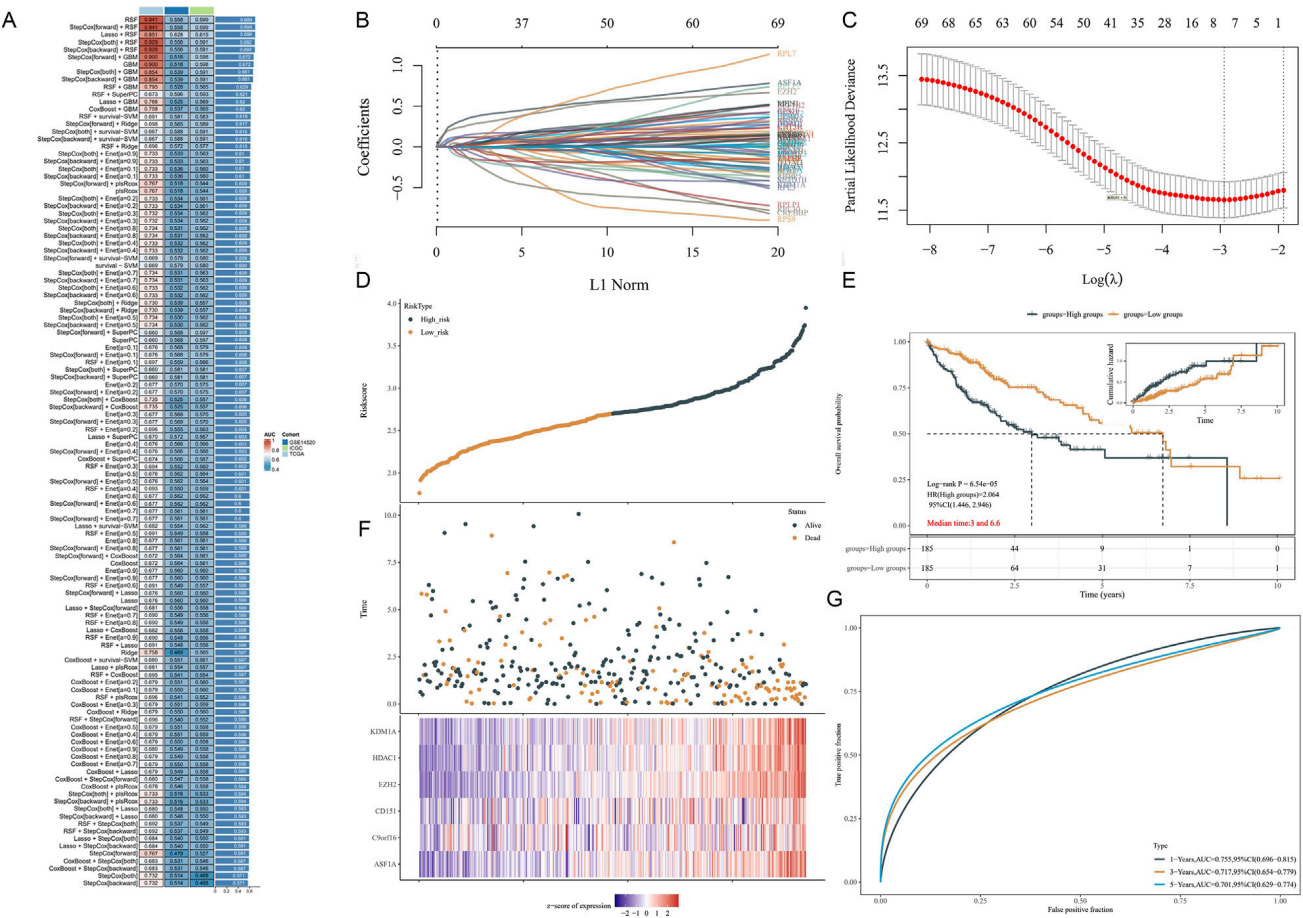
Risk score model based on 69 training genes constructed using Lasso regression method. **(A)** C-index calculated for 117 prediction models through 10-fold cross-validation framework across all validation datasets. **(B)** Lasso regression coefficient path plot for genes. **(C)** Lasso regression cross-validation deviance. *X*-axis represents log λ values, *Y*-axis represents deviance, red dots represent average deviance for each λ value, grey lines represent standard error of deviance, and vertical lines on *X*-axis represent optimal λ value. **(D)** Risk profile in the training set. **(E)** KM survival curves for high- and low-risk groups in the training set. **(F)** Distribution of Risk scores for each sample. **(G)** ROC curves for the training set.

Building upon the 45 HCC-HMRgenes, we incorporated 110 HM-related source genes, resulting in a total of 155 HMRgenes. To construct a robust prognostic model, we utilized the TCGA dataset as the training set and ICGC and GSE14520 as validation sets. We selected 69 HMRgenes common to all three datasets as input features (training genes) for machine learning. Using these 69 genes, we developed a consensus HMRS through integrated machine learning methods. Within a 10-fold cross-validation framework, we evaluated 117 different predictive models, assessing model performance by calculating the accuracy of each model across all datasets. Considering the comprehensive performance on the validation sets, we selected the Lasso + RSF model for HMRS construction (Figure 4A).

The Lasso regression coefficient path plot illustrated how the coefficients of the 69 genes shrink to zero as the L1 regularization penalty (λ value) increases, revealing the final selected model variables (Figure 4B). In Lasso regression, the cross-validation deviance plot determined the optimal λ value, which minimizes cross-validation error and provides the best model complexity (Figure 4C). Figure 4D displayed the risk score distribution of high- and low-risk samples in the training set, calculating based on the selected λ value. Kaplan-Meier survival curves demonstrated significant prognostic differences between high- and low-risk groups (*p* < 0.001), with median survival times of 3.0 and 6.6 years, respectively (Figure 4E). The risk score distribution plot illustrated the relationship between each sample’s score and survival status in the training set, with high-risk scores positively correlated with mortality events (Figure 4F). Subsequent ROC curve analysis evaluated the risk score model’s performance, yielding areas under the curve (AUC) of 0.76, 0.72, and 0.70 for 1-year, 3-year, and 5-year survival, respectively, indicating good predictive performance (Figure 4G). These results suggested that our constructed HMRS can accurately distinguish between high- and low-risk HCC patients, demonstrating strong prognostic predictive capability and robust model performance.

### 3.4 Performance evaluation and clinical relevance analysis of HMRS

**FIGURE 5:**
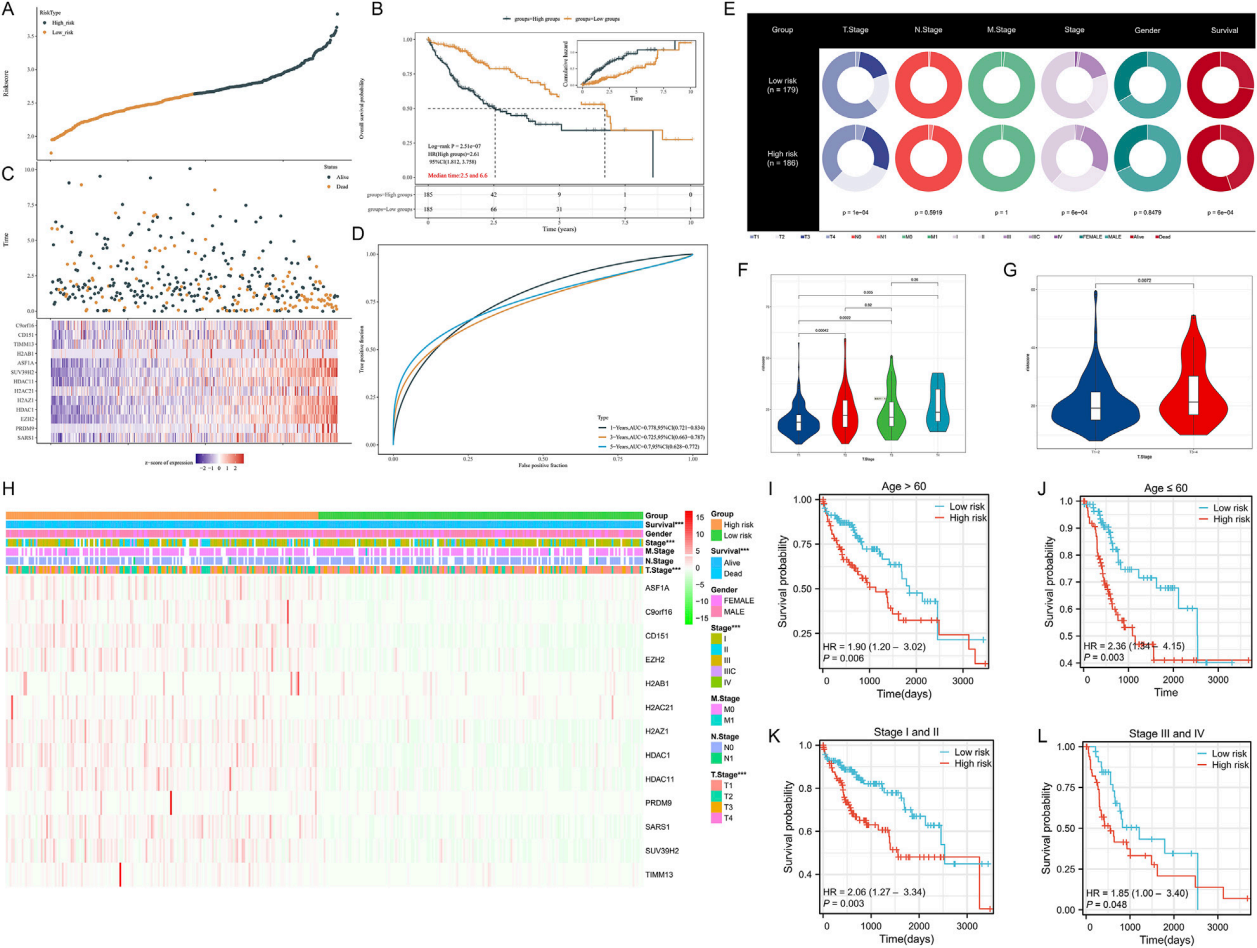
Performance evaluation and clinical relevance analysis of HMRS. **(A)** Survival analysis and predictive performance assessment of the risk score model based on 156 HMRgenes. **(B)** Distribution of HMRS low- and high-risk patients across different clinical features. **(C)** Violin plot of HMRS scores for patients at different T stages. **(D)** Heatmap analysis of model-selected gene expression and clinical features in high- and low-risk patient groups. **(E,F)** Kaplan-Meier survival curves after age stratification. **(G,H)** Kaplan-Meier survival curves after clinical stage stratification. **(I,J)** Survival analysis of patients stratified by age over 60 and under 60. **(K,L)** Survival analysis of patients stratified by early stage (I-II) and advanced stage (III-IV).

To further enhance the applicability and generalizability of the model, we expanded our analysis from the initial 69 training genes to include all 155 HMRgenes for retraining (Figure 5A). This expansion resulted in improved AUC values, with 1-year, 3-year, and 5-year AUCs reaching 0.78, 0.73, and 0.70, respectively.

We then investigated the distribution and performance of HMRS across different clinical feature subgroups. Figure 5B presents the distribution of HMRS low- and high-risk patients in terms of overall survival (OS), T stage, N stage, M stage, clinical stage, and gender. Notably, there was a significant difference between the two groups in T stage (*p* < 0.05). To further validate this finding, we used violin plots to visually demonstrate the differences in risk scores among patients at different T stages (Figure 5C). The results showed that patients at T3-4 stages had significantly higher risk scores than those at T1-2 stages (*p* = 0.0072). At the gene expression level, Figure 5D illustrates that the gene variables ultimately selected for the model were generally upregulated in the high-risk group. This result provides important clues about the biological basis of HMRS.

To assess the predictive stability of HMRS across different clinical contexts, we conducted stratified analyses. Figures 5E, F demonstrate that after age stratification, the survival rate of the high-risk group remained significantly lower than that of the low-risk group. Similarly, Figures 5G, H show that after stratification by clinical stage, patients in the high-risk group still had poorer survival prognoses. These results strongly support the potential of HMRS as an independent prognostic factor.

To further evaluate the predictive efficacy of HMRS across different age groups and disease stages, we conducted stratified analyses. In terms of age stratification, both patients aged over 60 (Figure 5I) and under 60 (Figure 5J) in the high-risk group showed significantly lower survival rates compared to the low-risk group (HR = 1.90 and 2.36, respectively, *p* < 0.05). Similarly, in the disease stage stratification analysis, patients in the high-risk group demonstrated poorer survival prognosis in both early stages (Stage I and II, Figure 5K) and advanced stages (Stage III and IV, Figure 5L) (HR = 2.06 and 1.85, respectively, *p* < 0.05). These results further confirm the potential of HMRS as an independent prognostic factor and demonstrate that the model maintains good predictive value across patient populations with different clinical characteristics.

### 3.5 Establishment and validation of nomogram integrating clinical features

**FIGURE 6:**
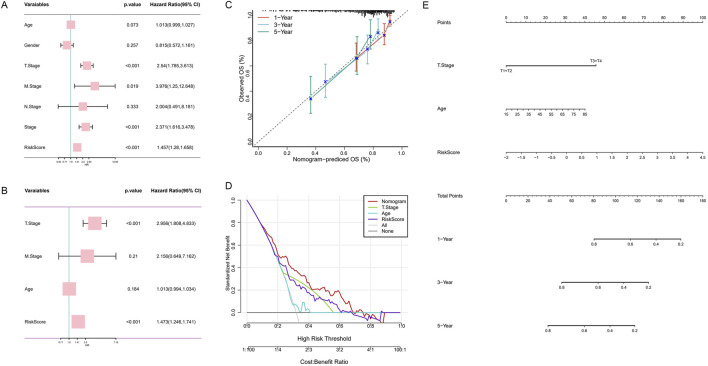
Construction and validation of a prognostic nomogram model integrating HMRS and clinical features. **(A)** Univariate analysis of the clinical characteristics and HMRS for OS. **(B)** Multivariate analysis. **(C)** Calibration curve of the nomogram for 1, 3, and 5-year OS. **(D)** Decision curve analysis showing the standardized net benefit by applying the nomogram and other clinical characteristics. **(E)** Construction of the nomogram based on the HMRS and clinical characteristics.

To assess the potential of HMRS as an independent prognostic factor for HCC, we conducted a comprehensive analysis of the impact of age, gender, TNM staging, clinical staging, and HMRS on overall survival (OS) in the TCGA-HCC cohort. Univariate Cox regression analysis (Figure 6A) revealed that age, T stage, M stage, and HMRS were significant prognostic factors for OS in the TCGA-HCC cohort (*p* < 0.1). Subsequent multivariate Cox regression analysis (Figure 6B) further confirmed the status of T stage and HMRS as independent prognostic indicators (*p* < 0.001).

Based on clinical experience and Cox regression analysis results, we selected T stage and age as key clinical features and integrated them with HMRS to construct a comprehensive prognostic nomogram (Figure 6E). In this nomogram, the point plot adjacent to each variable visually demonstrates its contribution to the predictive model, reflecting the strength of its association with survival prediction.

To validate the predictive accuracy of the nomogram, we plotted calibration curves (Figure 6C). The results showed that the nomogram-predicted 1-year, 3-year, and 5-year OS closely aligned with actual observed values, confirming the model’s reliability. Furthermore, decision curve analysis (Figure 6D) indicated that within a specific high-risk threshold range, the decision-making strategy based on the nomogram could achieve higher standardized net benefits compared to using other clinical features alone. This finding highlights the potential advantages of our constructed comprehensive prognostic model in clinical decision-making.

### 3.6 Transcriptomic characteristics analysis of different HMRS patient groups

To further investigate the molecular mechanisms underlying the correlation between HMRS and HCC prognosis, we conducted GSEA and GSVA analyses. These analyses revealed differences in biological processes and pathway activities associated with high and low HMRS score patient groups.

**FIGURE 7:**
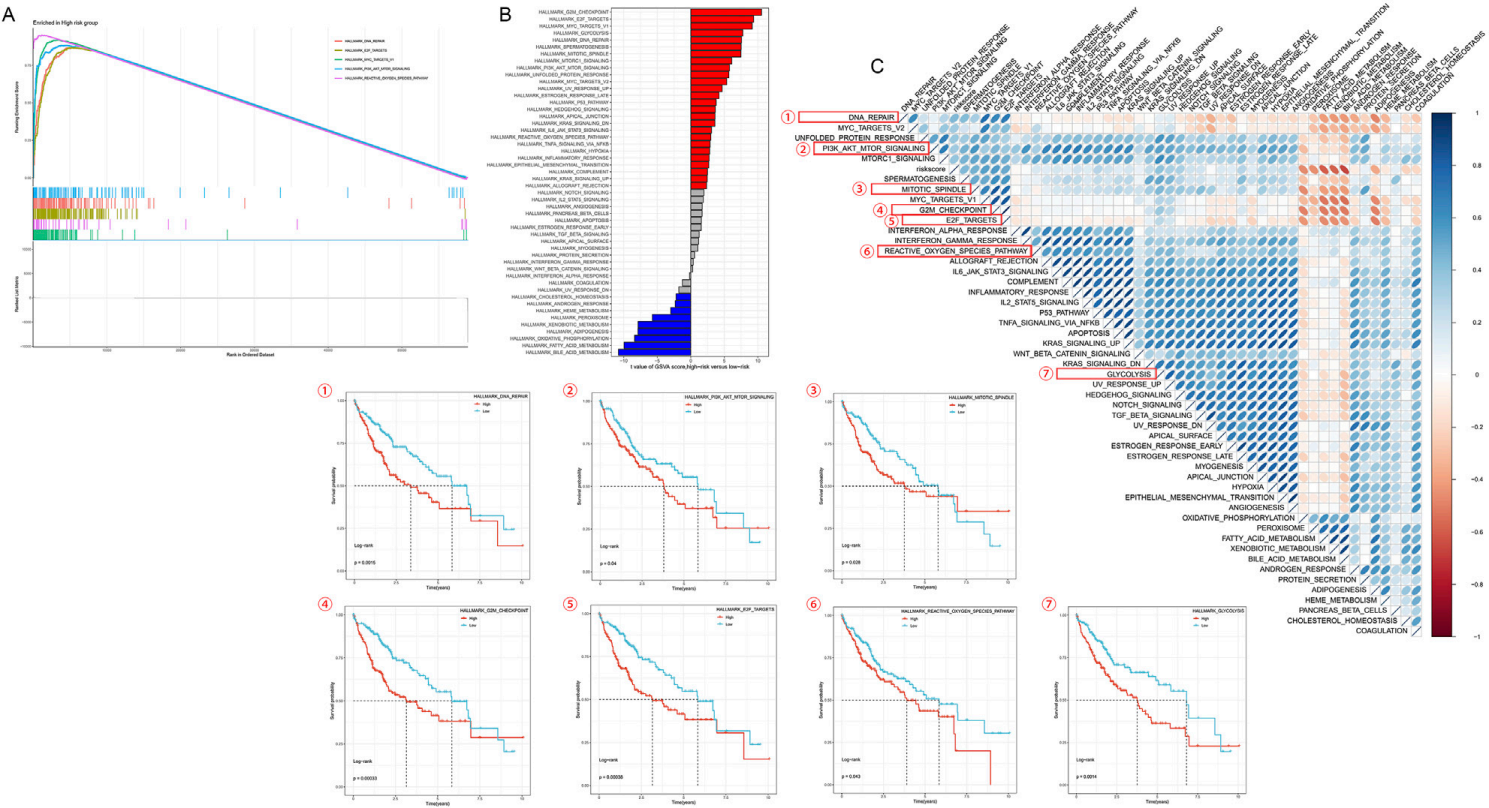
Transcriptomic characteristics of various HMRS patients. **(A)** GO terms enriched by GSEA analysis. **(B)** Differences in KEGG analysis between the high- and low-risk groups scored by GSVA. **(C)** Correlation between the risk score and hallmark pathway activities scored by GSVA. Kaplan-Meier survival curves respectively show survival differences between high and low expression groups for reactive oxygen species pathway, mitotic spindle, DNA repair, G2M checkpoint, E2F target genes, glycolysis, and PI3K/AKT/mTOR signaling pathway.

Figure 7A’s GSEA analysis uncovered GO pathways enriched in different HMRS groups. Figure 7B provides GSVA scores for KEGG pathways, further enhancing our understanding of pathway activities related to HMRS scores. Results showed that compared to the low-risk group, the high-risk group significantly enriched multiple gene sets associated with cell cycle, DNA repair, and metabolism. Notably, DNA repair, E2F target genes, MYC target genes, PI3K/AKT/mTOR signaling pathway, and reactive oxygen species pathway were significantly upregulated in the high-risk group.

The heatmap based on GSVA scores further confirmed significant differences between high- and low-risk groups. The heatmap displayed differential expression patterns of multiple signaling pathways and biological processes between the two groups, consistent with GSEA results. We selected several pathways that showed significance in both GSEA and GSVA analyses for survival analysis based on expression levels. Patients with high expression of reactive oxygen species pathway, mitotic spindle, DNA repair, G2M checkpoint, E2F target genes, glycolysis, and PI3K/AKT/mTOR signaling pathway showed significantly reduced survival rates (Figure 7C).

### 3.7 Mutation spectrum analysis of HM genes

**FIGURE 8:**
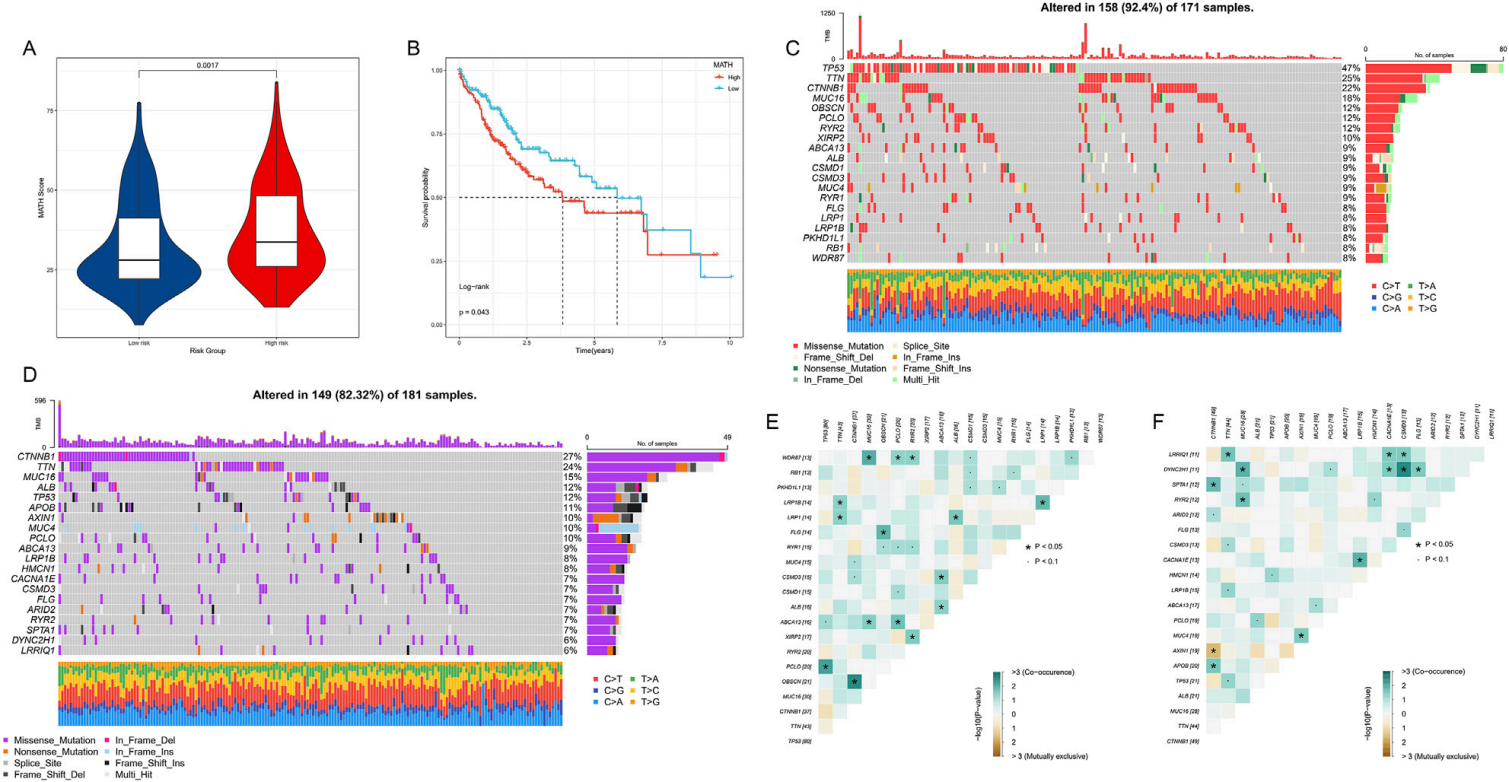
**(A)** MATH scores of high- and low-risk HCC patient groups. **(B)** Kaplan-Meier survival curves based on MATH scores. **(C)** Oncoplot of gene mutations in high-risk HCC patient group. **(D)** Oncoplot of gene mutations in low-risk HCC patient group. **(E,F)** Co-occurrence and mutual exclusivity analysis of the top 20 most mutated genes in high- and low-risk groups. Heatmap colors indicate relationships between gene pairs, asterisks denote statistical significance levels.

We conducted a comprehensive analysis of mutation patterns in histone modification genes among HCC patients, revealing significant differences between high- and low-risk groups. The MATH score was significantly higher in the high-risk group compared to the low-risk group (*p* = 0.0017), indicating greater tumor heterogeneity in the high-risk group (Figure 8A). Kaplan-Meier survival analysis based on MATH scores showed that patients with high MATH scores had worse prognoses (*p* = 0.043, Figure 8B), further confirming the association between tumor heterogeneity and prognosis.

We observed distinct gene mutation spectra between high- and low-risk groups (Figures 8C, D). In the high-risk group, TP53 had the highest mutation frequency (47%), followed by TTN (25%) and CTNNB1 (22%). In contrast, CTNNB1 had the highest mutation frequency in the low-risk group (27%), followed by TTN (24%) and MUC16 (15%). Notably, TP53 mutation frequency was significantly lower in the low-risk group (12%). In the high-risk group, we observed significant co-occurrence of TP53 mutations with several genes, including TTN, CTNNB1, and MUC16 (Figure 8E). In the low-risk group, CTNNB1 mutations appeared more independent from other gene mutations (Figure 8F).

### 3.8 HMRS-related immune landscape in HCC

**FIGURE 9:**
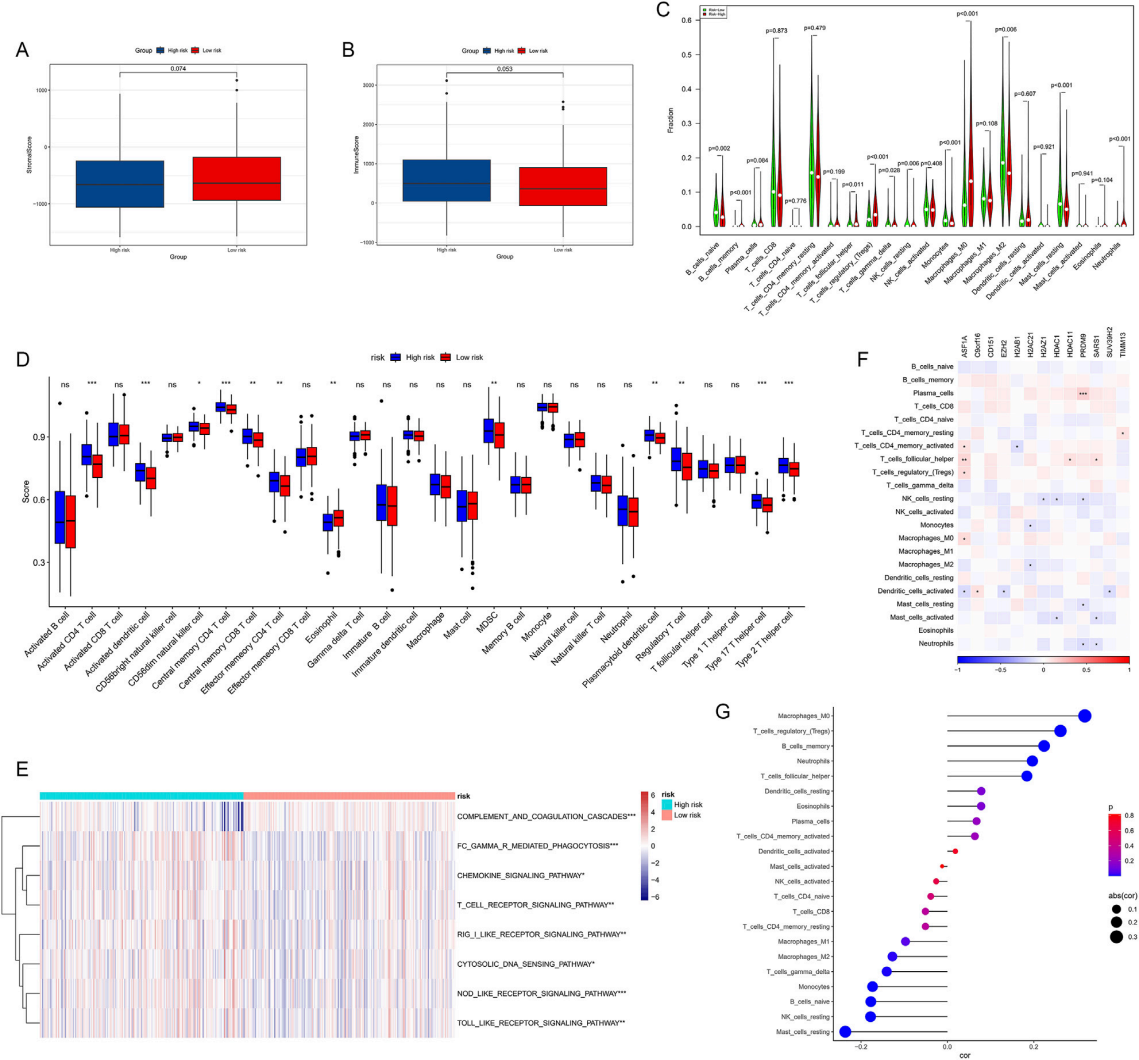
Correlations between immune microenvironment, immune characteristics, and HMRS. **(A, B)** The StromalScore and immune score were applied to quantify the different immune statuses between the high- and low-risk groups. **(C)** Abundance of each TME infiltrating cell type in high- and low-risk groups calculated using the CIBERSORT algorithm. **(D)** Infiltrating cell abundance calculated using quantitative scoring schemes for 28 immune phenotypes. **(E)** Immune-related pathways' activity showing significant differences between high- and low-risk groups. **(F)** Heatmap showing correlations between key HMs genes and 22 immune cell subgroups. **(G)** Dot plot showing correlations between risk scores and 22 immune cell subgroups.

We conducted a comprehensive analysis of the immune microenvironment in high- and low-risk HCC groups, including stromal scores, immune scores, immune cell infiltration, and related pathway analyses. Although differences in stromal scores (Figure 9A, *p* = 0.074) and immune scores (Figure 9B, *p* = 0.053) between high- and low-risk groups did not reach statistical significance, the high-risk group showed a trend towards lower scores in both metrics, suggesting that high-risk HCC may have weaker immune responses and stromal components.

To further analyze differences in specific immune cell infiltration between high- and low-risk groups, we used the CIBERSORT algorithm to calculate the abundance of each TME infiltrating cell type in both groups (Figure 9C). We found that memory B cells, follicular helper T cells, regulatory T cells, gamma delta T cells, M0 macrophages, and neutrophils were more abundant in the high-risk group, while naive B cells, resting NK cells, monocytes, M2 macrophages, and resting mast cells were more abundant in the low-risk group.

Subsequently, we quantified scores for 28 immune cell phenotypes (Figure 9D). Most immune cells showing significant differences had higher expression levels in the high-risk group, with only eosinophils showing higher expression in the low-risk group. Furthermore, using the ssGSEA algorithm, differences in immune-related pathway activities between high- and low-risk groups were demonstrated (Figure 9E). Several immune-related pathways, including complement and coagulation cascades, Fc-γ receptor-mediated phagocytosis, chemokine signaling pathway, and T cell receptor signaling pathway, were significantly activated in the high-risk group.

We then investigated the associations between infiltrating cells in the TME and the eight genes used to construct the HMRS (Figure 9F), revealing correlations between specific immune cell subgroups and gene expression patterns in the HMRS. For example, EZH2 showed positive correlations with various T cell subsets but negative correlations with B cells. These results suggest that HMs genes may participate in HCC progression by regulating immune cell infiltration.

Figure 9G displays the correlations between risk scores and 22 immune cell subgroups. The results indicate that risk scores are significantly positively correlated with Macrophages M0, memory B cells, and regulatory T cells, while negatively correlated with other cells (such as resting mast cells and resting NK cells). These findings collectively point to HMRS as an effective tool for quantifying the immune status of HCC patients, suggesting significant differences in immune landscape characteristics among patients with different risk levels.

### 3.9 Drug sensitivity prediction and HPA validation

**FIGURE 10:**
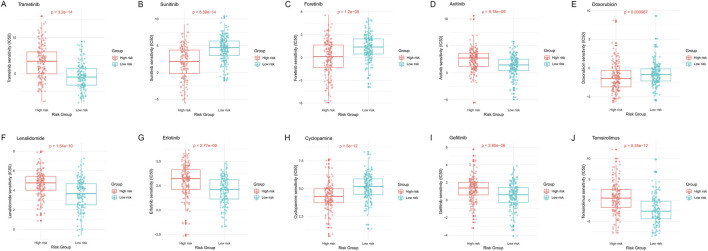
Distribution of IC50 scores for drugs in high- and low-risk groups defined by HMRS.

In Figure 10, through analysis of the GDSC database, we calculated IC50 values for commonly used drugs in HCC treatment across different cancer cell lines. Specifically, significant differences in IC50 values were observed between different risk groups for many drugs including Trametinib, Sunitinib, Foretinib, Axitinib, Doxorubicin, Lenalidomide, Erlotinib, Cyclopamine, Gefitinib, and Temsirolimus (Figures 10A-J). This emphasizes the potential value of these gene expression levels in predicting HCC patients' responses to specific drugs.

### 3.10 Experiment validation

**FIGURE 11:**
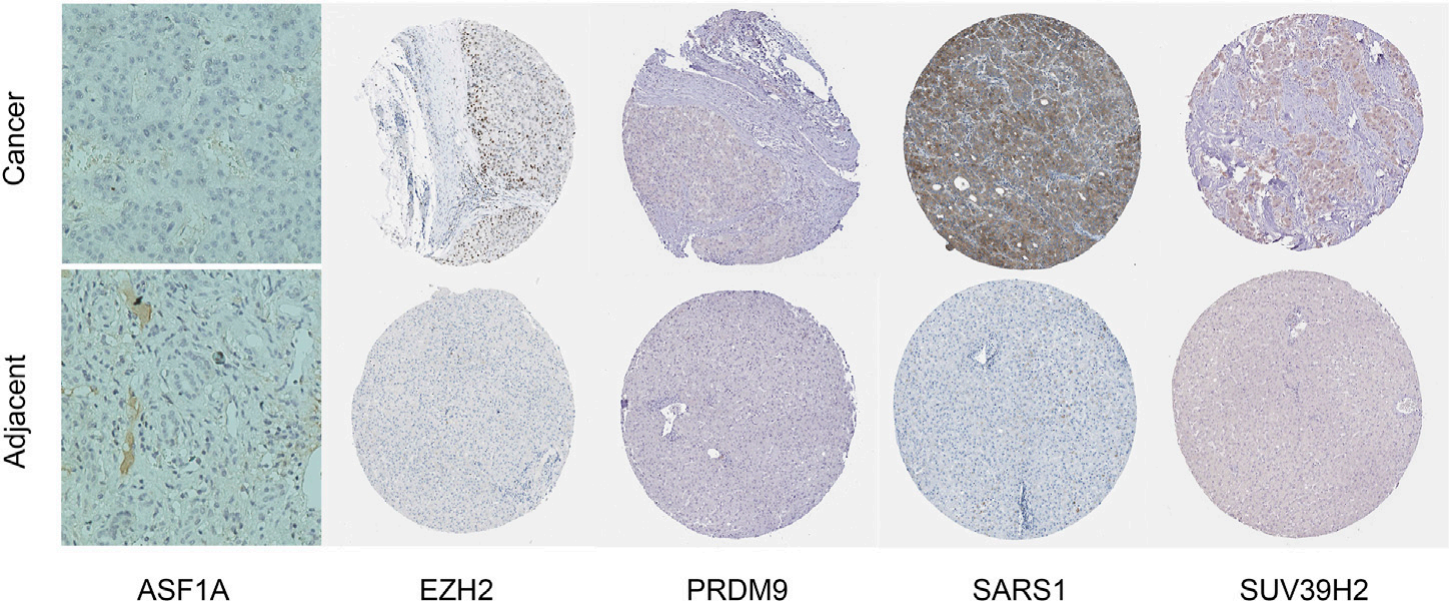
IHC staining of ASF1A, EZH2, PRDM9, SARS1, and SUV39H2 in HCC tissues and adjacent normal liver tissues. The upper row displays the expression of each gene in HCC tissues, while the lower row shows the corresponding adjacent normal tissues.

We performed IHC analysis to examine the expression of the five genes with the highest weights in our model (ASF1A, EZH2, PRDM9, SARS1, and SUV39H2). The results demonstrated that all five genes exhibited significantly upregulated expression patterns in HCC tissues compared to adjacent normal tissues (Figure 11). This validation supports the hypothesis that their increased expression may be associated with the initiation, progression, or maintenance of HCC.

## 4 Discussion

This study systematically explored the role and clinical significance of HMs in HCC by integrating multi-omics data and advanced computational methods. We first used single-cell RNA sequencing technology to reveal cellular heterogeneity in the HCC microenvironment, identifying six major cell clusters and analyzing differences in histone modification activity across different cell types. Subsequently, through WGCNA, we identified gene modules closely related to HMs. By summarizing and processing the corresponding differentially expressed genes, module genes, and single-cell characteristic genes, we visualized the HCC-related regulatory network of HMs. Based on these findings, we continued to construct a novel prognostic prediction model based on histone modification features using these genes. This model demonstrated good predictive ability in both the TCGA dataset and external independent datasets (GSE14520 and ICGC), providing strong evidence for risk stratification of HCC patients. Through further functional enrichment analysis, including GSEA and GSVA, we further elucidated key biological pathways associated with HMs. Additionally, we explored associations between HMs and gene mutations, immune microenvironment, and drug sensitivity, revealing the comprehensive and multifaceted role of HMs in HCC occurrence, development, and treatment response.

HMRS, as a novel prognostic marker, demonstrated significant potential as an independent prognostic factor in this study. Multivariate Cox regression analysis results showed that even when considering traditional clinical factors such as age, gender, and TNM staging, HMRS maintained significant prognostic predictive ability (*p* < 0.001). This finding highlights that HMRS captures important biological information not fully reflected by existing clinical indicators. The nomogram model integrating HMRS with key clinical features further improved the accuracy and clinical utility of prognostic prediction. Calibration curves showed high concordance between predicted 1-year, 3-year, and 5-year survival rates and actual observed values, while decision curve analysis confirmed that decision strategies based on the nomogram could achieve higher standardized net benefits within specific high-risk threshold ranges. This integrated approach not only improved prediction accuracy but also provided clinicians with an intuitive, user-friendly decision-making tool, facilitating individualized management of HCC patients. Compared to existing clinical staging systems, HMRS has distinct advantages in reflecting the molecular heterogeneity of HCC. Traditional TNM staging is mainly based on anatomical features of tumors and struggles to fully reflect tumor biological behavior and molecular characteristics. In contrast, HMRS, based on gene expression patterns related to HMs, can better capture the molecular biological properties of tumors. This molecular-level stratification not only more accurately predicts patient prognosis but may also provide guidance for targeted therapy and immunotherapy selection. For example, our study found that high-risk HMRS patients may be more sensitive to certain targeted drugs (such as Trametinib and Sunitinib), providing possibilities for HMRS-based individualized treatment decisions. However, it is worth noting that although HMRS shows superior predictive performance, it cannot completely replace existing clinical staging systems. Instead, HMRS should be viewed as a powerful complement to existing systems, and the combination of the two may provide more comprehensive and precise guidance for the comprehensive assessment and management of HCC patients.

Besides, HMRS, as a prognostic marker based on histone-related genes, not only reflects the molecular characteristics of HCC but also reveals the complex interactions between tumor evolution and the immune microenvironment. Our GSEA and GSVA analyses show that the activation of pathways such as DNA repair, cell cycle, and PI3K/AKT/mTOR in the high-risk HMRS group forms a seemingly contradictory but highly synergistic biological process with the activation of immune cell infiltration and immune-related pathways.

This apparent contradiction may reflect a concept: “epigenetic-mediated immune evasion” (Cacan, 2017). Specifically, the abnormal activation of DNA repair pathways in the high-risk group may not just be a mechanism to maintain genomic stability, but more likely a strategy for tumor cells to actively regulate their antigen expression profile. Through frequent DNA repair processes, tumor cells may dynamically adjust their neoantigen load, thereby evading immune surveillance. This hypothesis can explain why the high-risk group simultaneously exhibits higher immune cell infiltration and poorer prognosis (Germano et al., 2017; He et al., 2022).

The high tumor heterogeneity revealed by the mutation spectrum analysis of HMs genes may be a direct result of this “epigenetic-mediated immune evasion”. More frequent TP53 mutations in the high-risk group not only affect cell cycle regulation but may also influence the immunogenicity of tumor cells by altering global chromatin states (Wang et al., 2023; Nel et al., 2024). This links epigenetic regulation, genomic instability, and immune evasion, providing a new framework for understanding HCC progression.

The activation patterns of different signaling pathways in high- and low-risk HMRS groups, especially metabolism-related pathways (such as oxidative stress response), may play a key role in shaping the immune microenvironment. With the advancement of current technologies, numerous integrated studies on metabolism-immune-genetics have emerged (Ding et al., 2024; Fok et al., 2019). In this regard, we propose a new concept: the “metabolism-immune-epigenetic axis”. In this model, the metabolic reprogramming of tumor cells (as observed in the high-risk group) not only supports rapid proliferation but may also directly regulate the function and epigenetic state of local immune cells by producing specific metabolites (such as lactate, 2-hydroxyglutarate, etc.). This regulation may be bidirectional: changes in the metabolic state of immune cells may in turn affect the epigenetic profile of tumor cells, forming a complex feedback loop (Phan et al., 2017).

The differences in immune cell infiltration between high- and low-risk HMRS groups may reflect the dynamic balance of this “metabolism-immune-epigenetic axis”. For example, the higher infiltration of memory B cells and regulatory T cells observed in the high-risk group may be the result of tumor cells selectively recruiting and maintaining these immune cell subsets that favor tumor growth through specific epigenetic modification patterns. This selectivity may be achieved by regulating the expression of specific chemokines or cytokines, whose genes may be key epigenetic regulatory targets captured by HMRS (Cao and Yan, 2020; Hogg et al., 2020).

Based on these observations, we propose that HMRS may have unique value in predicting immune therapy response. Traditional immune therapy prediction markers (such as PD-L1 expression or tumor mutation burden) may not capture this complex “epigenetic-mediated immune evasion” mechanism. HMRS, as a marker integrating information from multiple levels, may more accurately reflect the tumor’s “immune evasion potential”. For example, high-risk HMRS patients may require a combined treatment strategy targeting epigenetic regulation (such as DNA methylation inhibitors or histone deacetylase inhibitors) and immune checkpoint inhibitors to reshape the tumor immune microenvironment and enhance the effects of immunotherapy (Prasanna et al., 2018).

Although this study has made significant progress in revealing the importance of histone-related genes in HCC, there are still some limitations. Firstly, our analysis is mainly based on public datasets, which may not fully represent the heterogeneity of all HCC patients. Secondly, although our HMRS model has shown good predictive ability in multiple independent cohorts, it lacks prospective clinical validation. Additionally, despite our extensive bioinformatics analysis, we lack laboratory validation to confirm the observed molecular mechanisms (Wu et al., 2023). Finally, our drug sensitivity analysis is based on *in vitro* cell line data, which may not fully reflect the complex microenvironment of tumors *in vivo*.

This study successfully constructed a prognostic model (HMRS) based on histone-related genes through multi-omics integrated analysis, providing a new perspective for precision diagnosis and treatment of HCC. Our findings not only reveal the importance of HMs in HCC but also provide a basis for potential therapeutic targets and individualized treatment strategies. Future research directions should include: 1) Validating the predictive value of HMRS in larger-scale prospective clinical trials; 2) Conducting in-depth functional experiments to elucidate the specific mechanisms of key HMs genes in HCC progression; 3) Developing new treatment strategies targeting HMs (e.g., histone deacetylase inhibitors) or subsequent pathways (e.g., targeted therapies for upregulated pathways like PI3K/AKT/mTOR in high-risk groups) based on our findings, and evaluating their effects in preclinical models; 4) Exploring the combined application of HMRS with existing treatment methods (e.g., immunotherapy targeting regulatory T cells) to improve the overall efficacy of HCC treatment. Through these efforts, we hope to further advance individualized treatment for HCC and ultimately improve patient prognosis.

## Data Availability

The original contributions presented in the study are included in the article/supplementary material, further inquiries can be directed to the corresponding authors.
